# Genome-wide transcriptional response of primary alveolar macrophages following infection with porcine reproductive and respiratory syndrome virus

**DOI:** 10.1099/vir.0.2008/003244-0

**Published:** 2008-10

**Authors:** Sem Genini, Peter L. Delputte, Roberto Malinverni, Maria Cecere, Alessandra Stella, Hans J. Nauwynck, Elisabetta Giuffra

**Affiliations:** 1 Parco Tecnologico Padano – CERSA, Via A. Einstein, 26900 Lodi, Italy; 2 Department of Virology, Parasitology, and Immunology, Faculty of Veterinary Medicine, Ghent University, Merelbeke, Belgium

## Abstract

Porcine reproductive and respiratory syndrome is a major cause of economic loss for the swine industry worldwide. Porcine reproductive and respiratory syndrome virus (PRRSV) triggers weak and atypical innate immune responses, but key genes and mechanisms by which the virus interferes with the host innate immunity have not yet been elucidated. In this study, genes that control the response of the main target of PRRSV, porcine alveolar macrophages (PAMs), were profiled *in vitro* with a time-course experiment spanning the first round of virus replication. PAMs were obtained from six piglets and challenged with the Lelystad PRRSV strain, and gene expression was investigated using Affymetrix microarrays and real-time PCR. Of the 1409 differentially expressed transcripts identified by analysis of variance, two, five, 25, 16 and 100 differed from controls by a minimum of 1.5-fold at 1, 3, 6, 9 and 12 h post-infection (p.i.), respectively. A PRRSV infection effect was detectable between 3 and 6 h p.i., and was characterized by a consistent downregulation of gene expression, followed by the start of the host innate immune response at 9 h p.i. The expression of beta interferon 1 (*IFN*-*β*), but not of *IFN*-*α*, was strongly upregulated, whilst few genes commonly expressed in response to viral infections and/or induced by interferons were found to be differentially expressed. A predominance of anti-apoptotic transcripts (e.g. interleukin-10), a shift towards a T-helper cell type 2 response and a weak upregulation of tumour necrosis factor-*α* expression were observed within 12 h p.i., reinforcing the hypotheses that PRRSV has developed sophisticated mechanisms to escape the host defence.

## INTRODUCTION

Porcine reproductive and respiratory syndrome is a major cause of economic loss for the swine industry worldwide (Neumann *et al.*, 2005) and causes high mortality of nursery piglets, reproductive failure in sows, respiratory distress in pigs of all ages and influenza-like symptoms in grow/finish swine (Mengeling & Lager, 2000; Nodelijk, 2002). The aetiological agent is porcine reproductive and respiratory syndrome virus (PRRSV), belonging to the family *Arteriviridae* with an enveloped, positive-stranded RNA genome of about 14.5 kb (Snijder & Meulenberg, 1998).

A typical hallmark of PRRSV is that it causes an acute viraemic phase (up to 14 days post-inoculation) during which the virus can be detected in serum and all susceptible organs (Beyer *et al.*, 2000; Duan *et al.*, 1997b). This acute phase is followed by virus elimination from serum and most organs, and by persistent replication in tonsils, lungs and some lymph nodes (Allende *et al.*, 2000; Rowland *et al.*, 2003; Wills *et al.*, 2003). This prolonged replication does not represent a true persistent infection, as all animals clear the virus by 6 months after inoculation, thus indirectly showing that the immune system is capable of finally dealing with the virus, although not efficiently. Because of this persistent nature of PRRSV infections, numerous studies have analysed the immune responses that may control PRRSV infections or that may be altered by PRRSV (reviewed by Lopez & Osorio, 2004; Mateu & Diaz, 2007; Murtaugh *et al.*, 2002).

The PRRSV-specific humoral immunity is generally characterized by a strong, non-neutralizing antibody response, which is detected from 5–6 days post-infection (p.i.). In contrast, induction of neutralizing antibodies is severely delayed (starting at 3–4 weeks p.i.) and their levels remain low (Lopez & Osorio, 2004); antibodies were shown to be ineffective in eliminating PRRSV-infected macrophages in combination with complement (Costers *et al.*, 2006). Cellular immune responses against PRRSV infection are characterized by a late onset of lymphocyte proliferative responses (4 weeks p.i.) and the late appearance of gamma interferon (IFN-*γ*)-secreting cells (Meier *et al.*, 2003). Several studies have also shown weak and atypical innate immune responses, such as weak IFN-*α* responses and high induction of interleukin (IL)-10. This inadequate recognition of virus infection by the innate defence mechanisms could be responsible for the initially crippled immune response (Albina *et al.*, 1998; Buddaert *et al.*, 1998; Murtaugh *et al.*, 2002; Royaee *et al.*, 2004; Suradhat *et al.*, 2003; van Reeth *et al.*, 1999; Xiao *et al.*, 2004). The mechanism by which PRRSV interferes with innate immune responses has yet to be elucidated.

PRRSV has a highly specific tropism for cells of the monocyte/macrophage lineage, cells that are essential for immune function. *In vivo*, the virus mainly infects a subpopulation of differentiated macrophages that are present in tonsils, lungs and other lymphoid tissues (Beyer *et al.*, 2000; Duan *et al.*, 1997a, b). Besides macrophages, *in vitro* analysis of susceptible cells has identified cultivated monocytes and dendritic cells as potential targets, but their role during PRRSV infections *in vivo* remains to be established (Delputte *et al.*, 2007; Duan *et al.*, 1997a; Loving *et al.*, 2007; Teifke *et al.*, 2001; Voicu *et al.*, 1994; Wang *et al.*, 2007). Lung pathogenesis is another feature of PRRSV infections, and porcine alveolar macrophages (PAMs) are generally considered to be a major target for PRRSV.

The aim of this study was to gain insight into the putative mechanisms by which PRRSV can evade innate immunity, and consequently the adaptive response, using a genome-wide approach. A time-course gene expression profiling of PAMs infected *in vitro* with a reference strain (Lelystad) was conducted by utilizing an Affymetrix 24K Porcine Chip microarray. Collection of samples at different times during the infection cycle, from 1 h p.i. (virus entry) up to 12 h p.i. (virus release and cell death) allowed us to discriminate between changes in early and late gene expression during infection. Times later than 12 h p.i. were not analysed, as by that time PRRSV infection of macrophages has typically resulted in cell death.

## METHODS

### Cells and treatments.

Six 3-week-old hybrid piglets from a PRRSV- and porcine circovirus 2-negative herd of the Rattlerow–Seghers genetic line (a cross-breed between English Landrace, Belgian Landrace, Large White and a synthetic company Landrace) were injected daily with 1 ml enrofloxacin (5 % solution) and 1 ml lincospectin/spectinomycin (5 or 10 % solution) for 3 days to eliminate eventual bacterial pathogens. Two weeks later, the piglets were sacrificed. PAMs were collected by bronchoalveolar lavage and frozen in liquid nitrogen as described by Wensvoort *et al.* (1991).

**Fig. 1. f1:**
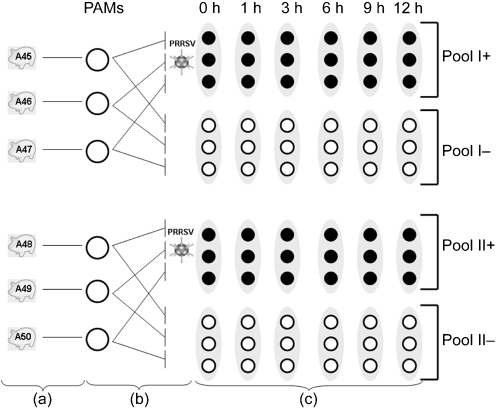
Schematic representation of the experimental design used in this study to challenge PAMs with PRRSV *in vitro*. PAMs were obtained from six piglets (a). Each PAM culture was split into two and infected with PRRSV or mock infected as a control (b). The total RNA from PAMs of each piglet was extracted at different time points (0, 1, 3, 6, 9 and 12 h p.i.). The RNA of three piglets was pooled (pools I and II) for the subsequent microarray and real-time analyses (c).

PAMs were thawed and cultured for 48 h before treatment as described previously by Delputte & Nauwynck (2004). One primary culture from each animal was split into two: one was infected at an m.o.i. of 10 with a 13th passage of PRRSV Lelystad virus (kindly provided by G. Wensvoort, Institute for Animal Science and Health, Lelystad, The Netherlands), which was semi-purified as described previously (Delputte & Nauwynck, 2004). The other culture was maintained as a control and was mock inoculated. The percentage of infected cells ranged between 60 and 70 % for all batches. Cells were collected at 1, 3, 6, 9 and 12 h p.i. in TRIzol (Invitrogen Life Technologies) for RNA extraction (Fig. 1).

### RNA extraction, reverse transcription, RNA labelling and cRNA hybridization.

Total RNA extraction from PAMs was performed using TRIzol following standard instructions (Invitrogen) and a clean-up was carried out using RNeasy columns (Qiagen). RNA quality was assessed by microcapillary electrophoresis on an Agilent 2001 Bioanalyser (Agilent Technologies) with RNA 6000 Nanochips. RNA was quantified by spectrophotometry (ND-1000; NanoDrop Technologies). Reverse transcription of 20 μg total RNA and synthesis of biotin-labelled cRNA with one round of amplification were carried out following the standard Affymetrix one-cycle protocol according to the manufacturer's instructions.

Transcriptional profiles were assessed using Affymetrix 24K GeneChip Porcine Genome Arrays (http://www.affymetrix.com/products/arrays/specific/porcine.affx). Based on previous evidence that sample pooling does not significantly affect the results of Affymetrix chip analysis (see, for example, Han *et al.*, 2004), three samples each from control and infected-cell cultures were pooled for each time point (Fig. 1), resulting in two control (pools I^−^ and II^−^) and two infected pools (pools I^+^ and II^+^).

Hybridization and scanning of the arrays were carried out according to standard Affymetrix protocols (Shen *et al.*, 2005) using a GeneChip Scanner 3000 7G.

### Microarray data analysis.

Signal intensities were evaluated using the GeneChip Operating Software algorithm (gcos version 1.4; Affymetrix). Raw data and statistical analyses were performed with GeneSpring version 7.3.1 software (Agilent). Normalization was performed per chip (normalized to 50th percentile) and per gene (normalized to the median).

A statistical analysis of variance (ANOVA) model was applied to the data and significance was declared accepting a false discovery rate (FDR) of 0.05. Fixed effects of time point and status (infected−non-infected cells) were included in the ANOVA model. A further cut-off threshold was applied based on a fold change of 1.5 between infected and control PAMs. Hierarchical clustering of the conditions was performed using Pearson's correlation coefficient (*r*) as a measure of similarity and the average linkage method as the clustering algorithm.

In order to test for the presence of outliers in the two pools, the transcriptional profiles of infected animals were analysed separately at the 3 h p.i. time point. A paired *t*-test (paired across pools by gene) was performed using the range of minimum and maximum corrected expression values within each pool for each gene. The test was applied (i) to the whole set of genes and (ii) to the subset of genes that appeared to be significant for differential expression in the general analysis. No significant difference was observed either for the whole set of genes included in the study or for the subset of differentially expressed genes.

The Database for Annotation, Visualization and Integrated Discovery (DAVID 2006; http://david.abcc.ncifcrf.gov/), an expanded version of the original web-accessible programs described by Dennis *et al.* (2003), was used to allocate transcripts with similar biological questions into the three gene ontology (GO) categories.

### Real-time PCR.

Quantitative real-time PCR analysis was conducted on ten selected swine transcripts and on the *ORF7* gene of PRRSV. Hypoxanthine phosphoribosyltransferase (HPRT1) was chosen as the reference gene because the amplifications of all control and infected samples showed very similar threshold cycle (Ct) values (data not shown). The transcript-specific primers were designed using ProbeFinder software (version 2.3) on the Roche website (https://www.roche-applied-science.com/sis/rtpcr/upl/adc.jsp) using standard settings for the human Universal Probe Library Set catalogue (see Supplementary Table S1, available with the online version of this paper).

Two micrograms of total RNA from pools I and II were reverse-transcribed using the Superscript II RT-PCR System (Invitrogen Life Technologies) and standard procedures. The real-time reaction mixture (total 20 μl) included 5 μl cDNA as template (diluted 1 : 50), 200 nM of each of the two primers (forward and reverse), 100 nM Roche probe and 1× master mix (Applied Biosystems). Real-time PCR was performed in 384-well optical plates using a Tecan Freedom EVO-150 liquid handling workstation (Tecan Trading) and an ABI 7900HT real-time PCR machine (Applied Biosystems) with the GeneAmp 7900HT sequence detection system software (PerkinElmer).

A control cDNA dilution series (1 : 50, 1 : 100, 1 : 500 and 1 : 5000) was created for each transcript to establish a standard curve for each plate; real-time reactions of the same pools described for the microarray analysis were performed in triplicate. Briefly, the log input amount of the standard curve was plotted against the output Ct values; all amplifications had a slope of between −3.48 and −2.99 and were accepted as quantitative. The log input amount of each sample was then calculated according to the formula (Ct−*b*)/*m*, where *b* is the *y*-intercept and *m* is the slope. The log input amount was converted to input amount according to the formula 10^log input amount^ and triplicate input amounts were averaged for each sample. The mean input amount of each gene was normalized to the mean input amount of HPRT1. A *t*-test (with thresholds for statistical significance set to 0.1 and 0.05) was applied to each gene to verify whether the difference between control and infected macrophages at each time point was significant.

Pearson's correlation coefficient (*r*) was calculated for each gene on the normalized data to quantify the consistency between microarray experiments and real-time PCR.

### Microarray data.

The data of the microarray analysis were deposited in the ArrayExpress repository (http://www.ebi.ac.uk/arrayexpress) with ArrayExpress accession number ×10−MEXP-1350, following the guidelines of the rationale of minimum information about a microarray experiment (MIAME) (Brazma *et al.*, 2001).

## RESULTS

### Microarray analysis

**Fig. 2. f2:**
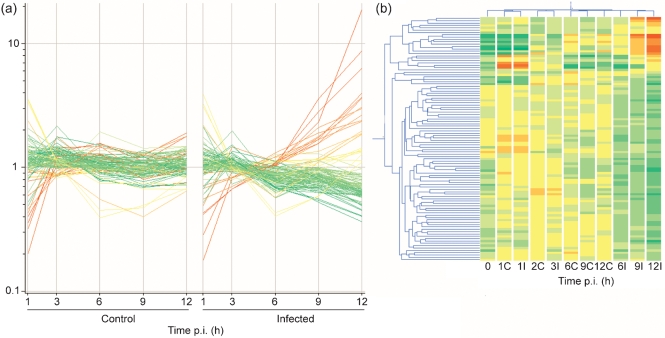
(a) Distribution of signal intensities of the 100 transcripts differentially expressed at 12 h p.i. over the period of infection. Left, control PAMs; right, infected PAMs. Each line represents a transcript. (b) Hierarchical clustering of the different time point conditions, based on the transcripts differentially expressed at 12 h p.i. in control (C) and infected (I) PAMs. Coloration in both figures refers to the condition of infected cells at 12 h p.i. and is directly proportional to the expression, ranging from red (high expression) to green (low expression).

ANOVA analysis (FDR=0.05) showed that 1409 genes were differentially expressed in macrophages after PRRSV infection. After applying a further filter of 1.5-fold change in expression, two, five, 25, 16 and 100 transcripts were differentially expressed at 1, 3, 6, 9 and 12 h p.i., respectively, compared with the controls at the same time points. Overall, the effect of PRRSV on the host transcription machinery was one of downregulation (115/148 transcripts). The differentially expressed transcripts were annotated based on a previous work (Tsai *et al.*, 2006) and are reported in Table 1. The distribution of signal intensities of the 100 differentially expressed transcripts at 12 h p.i. and the hierarchical clustering of controls and infected replicates for the five time conditions (plus the time 0) are shown in Fig. 2.

At early time points (1 and 3 h p.i.), the profiles of gene expression in the control and infected conditions were very similar and clustered together, i.e. only two (1 h p.i.) and five (3 h p.i.) transcripts were significantly altered. The expression profiles clearly changed between 3 and 6 h p.i., with greater differences detected at the later time points (9 and 12 h p.i.), when PRRSV has been shown to complete its replication (Halbur, 2001; Rossow *et al.*, 1995).

The 6 h p.i. time point was characterized by a consistent downregulation of gene expression in the infected cells. The 24 downregulated transcripts represented genes with functions related to RNA processing (*HNRPLL* and *NXT2*), regulation of biological processes (*ATF2*, *PTPRC*, *HIF1*-*α*, *DLC1* and *RB1CC1*) and signal transduction (*PLAA*, *PTPRC*, *HIF1*-*α*, *DLC1* and *GPR160*). Only one transcript, an RNA-dependent helicase (*DDX17*), was upregulated.

The 9 h p.i. time point was the only one at which most transcripts (11/16) were upregulated. These represented genes (*IFIT1*, *GBP1*, *USP18* and *cig5*) that encode accessory proteins related to the immune response, and in particular to the pro-inflammatory cytokine IFN-*β*, but also genes with a known anti-apoptotic function (*ADM* and *TNF*-*αIP3*).

At 12 h p.i., the downregulated transcripts were also largely predominant over the upregulated ones (80 vs 20, respectively). The latter confirmed the main pattern of anti-apoptotic and antiviral response already observed at 9 h p.i., with the addition of two new transcripts representing *TNF-α* and *IL-10*. The overall highest fold change (FC) was observed for *IFN-β* (FC=20.15 at 12 h p.i.), whilst the most downregulated transcript was *NP_060114* (FC=0.306 at 12 h p.i.). *NP_060114* corresponds to the human DRE1 protein, a member of the kelch-repeat family, which modulates host immune response to viral infection (Prag & Adams, 2003). *KHLX* belongs to the same family and also showed a consistent downregulation at 12 h (FC=0.654).

The GO analysis assigned the 100 differentially expressed transcripts at 12 h p.i. to 34 biological processes, five molecular functions and three cellular components (Table 2), with the best ranked biological processes (response to stimulus, response to stress and immune response) effectively representing the general pattern of cell response to infection. This was independently confirmed by assigning the same 100 transcripts to regulatory pathways using the Kyoto Encyclopedia of Genes and Genomes (KEGG) database. The most represented pathways were all related to the immune response and included mitogen-activated protein kinase (MAPK), JAK–STAT, natural killer cell-mediated cytotoxicity, T-cell receptor signalling, cytokine–cytokine receptor interaction and Toll-like receptor signalling (data not shown).

### Validation of the microarray data by real-time PCR

The genes tested by real-time PCR (see Supplementary Table S1) were selected to validate the microarray results and to confirm the involvement of key biological pathways. The set included the differentially expressed genes *IFN*-*β*, *cig5*, *TNF*-*α*, *TNF*-*αIP3*, *IL-10*, *USP18* and *GRP58*, as well as three additional genes selected from recent literature (*IFN*-*α*, *IFN*-*αR1* and *sialoadhesin*), which were represented on the array but had not shown differential expression (Table 3). Pearson's correlation coefficient (*r*) showed that both the microarray and real-time PCR data were highly correlated for the genes modulated between 6 and 12 h p.i., with a high consistency between pools and with *r* values ranging from 0.95 (*IFN*-*β*) to 0.88 (*USP18* and *IL-10*) (Table 3). Only *GRP58*, shown by microarrays to be upregulated at 1 h p.i. but downregulated at 6 h p.i., showed inconsistencies between pools and a very poor *r* value.

**Fig. 3. f3:**
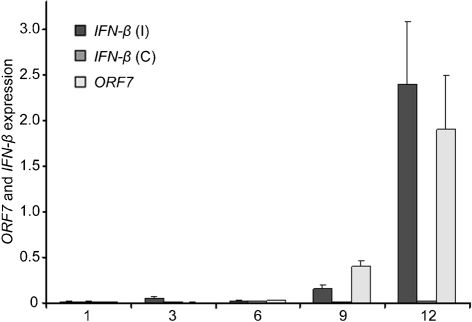
Comparison of the time-course expression of host *IFN*-*β* and PRRSV *ORF7* expression by real-time PCR in control (C) and infected (I) PAMs at five time points p.i. Results are shown as means±sd.

Real-time PCR confirmed that *IFN*-*β* was the most upregulated gene, whilst *IFN*-*α* was not differentially expressed between control and infected cells at 9 and 12 h p.i. Moreover, real-time PCR analysis of PAMs in an independent challenge experiment, with a different viral strain and lower m.o.i., confirmed that, at 24 h p.i., *IFN*-*β* was strongly induced whilst *IFN*-*α* was only slightly upregulated (data not shown). The expression of *IFN*-*β* increased together with the PRRSV titre, as affirmed by PRRSV *ORF7* gene expression (Fig. 3). Interestingly, real-time PCR at 3 h p.i. showed a small but significant peak in *IFN*-*β* and *IFN*-*α* expression, which was not detected by microarrays. *IFN*-*αR1* was confirmed not to be differentially expressed. Statistically significant differences in values of *sialoadhesin* expression were found between infected and control samples, but this was inconsistent between pools.

## DISCUSSION

The finding that the only gene to be upregulated at 6 h p.i. was a host RNA-dependent helicase (*DDX17*) indicates that PRRSV does not induce a generalized suppression of host gene transcription. This confirms and reinforces previous observations (Zhang *et al.*, 2000), showing enhanced production of a cellular helicase (RHIV-1) in macrophages in response to PRRSV infection. Other RNA viruses, such as poliovirus and vesicular stomatitis virus, replicating exclusively in the cytoplasm using virus-encoded RNA-dependent RNA polymerases and thus not requiring the host transcriptional apparatus, inhibit nuclear transcription of cellular RNA polymerases (Weidman *et al.*, 2003). This transcription shut-off not only allows the virus to evade cellular responses, but also may favour viral RNA replication by increasing the pool of free ribonucleotides in the cell. The complex expression pattern revealed by the microarrays might suggest that PRRSV blocks specific cellular transcription processes while upregulating others that are potentially beneficial for virus replication. By having a regulatory effect on cellular transcription, viruses may promote their own replication while interfering with the innate and adaptive immune responses that would result in their removal.

The downregulation of four genes encoding mitochondrial proteins (*NP_060530*, *SDHC*, *MRPL2* and *MRPL22*) at different time points (6, 9 and/or 12 h p.i.) might add up to the emerging role of mitochondria in antiviral immunity. The mitochondrial antiviral signalling protein MAVS is critical for the IFN-*β* signalling pathway in response to dsRNA, and is required for both TLR3-mediated and TLR3-independent signalling pathways, such as that triggered by the RNA helicase RIGI (Moore *et al.*, 2008; Xu *et al.*, 2005; Yoneyama *et al.*, 2004). RIGI is the product of *DDX58*, a member of the DEAD box family of RNA helicases that mediate nucleoside triphosphate-dependent unwinding of dsRNA and are involved in many diverse cellular functions (Lamm *et al.*, 1996). Intriguingly, the only upregulated gene found by microarrays at 6 h p.i. in PAMs (*DDX17*) belongs to the same family.

The atypical pattern of expression of innate immunity genes indicates that PRRSV has probably developed sophisticated mechanisms to control the antiviral response. Indeed, only a subset (*IFIT1*, *GBP1*, *USP18* and *TNF*-*αIP3*) of genes commonly modulated by pathogens in response to dsRNA and/or stimulated by IFN (Jenner & Young, 2005) were found to be upregulated by PRRSV at 9 and/or 12 h p.i. When the 1.5-fold change threshold was not applied after ANOVA analysis, this subset also included *CD44*, *PML*, *PRKRA*, *CCl4*, *CCl8* and *MT2A*. Upregulation of *USP18* has been observed previously in PAMs following PRRSV infection (Zhang *et al.*, 1999). The same study reported the upregulation of the antiviral gene *MX1*, but neither *MX1* nor *MX2* was found to be differentially expressed in the present investigation. Downregulation of *NRAMP2* at 12 h p.i. was consistent with the effects observed previously in humans after human immunodeficiency virus infection (reviewed by Jenner & Young, 2005).

Production of IFN-*α* and IFN-*β* is a well-known reaction of virus-infected cells; however, only the *IFN*-*β* gene was strongly upregulated by PRRSV in PAMs. The induction of *IFN*-*β* mRNA, but not *IFN*-*α* mRNA, has also been observed in monocyte-derived dendritic cells infected by PRRSV at 12 h p.i. (Loving *et al.*, 2007). Previous studies, both *in vitro* and *in vivo*, have also shown that PRRSV is a poor inducer or even a suppressor of IFN-*α* compared with other respiratory viruses (Albina *et al.*, 1998; Buddaert *et al.*, 1998; Miller *et al.*, 2004; van Reeth *et al.*, 1999). Blocking IFN-*α* production clearly is beneficial for PRRSV replication, as IFN-*α* can efficiently block replication when present during infection (Delputte *et al.*, 2007; Loving *et al.*, 2007). IFN-*β* can also protect macrophages against PRRSV infection (Overend *et al.*, 2007), but it has been suggested that IFN-*β* alone may be not sufficient to trigger the adaptive immune response (Loving *et al.*, 2007). A recent report has shown that *in vitro* stimulation of monocytes and macrophages with IFN-*α* induces expression of sialoadhesin, the main PRRSV receptor in PAMs, and that treatment with IFN-*α* before inoculation strongly increases PRRSV infection of monocytes (Delputte *et al.*, 2007). In agreement with this, in this study neither the gene encoding sialoadhesin nor that encoding IFN-*α*R1 (IFN receptor 1) showed consistent differential expression in infected cells.

Despite previous evidence that IFN-*β* expression by infected cells mediates and potentiates apoptosis (Tanaka *et al.*, 1998), the present study showed a predominance of transcripts leading to prolonged cell survival within 12 h of infection (both upregulation of anti-apoptotic transcripts and downregulation of pro-apoptotic genes). Upregulation was observed for *IL-10*, *ADM* and *TNF*-*αIP3*. IL-10 has been demonstrated to protect cells against apoptosis (Sieg *et al.*, 1996; Zhou *et al.*, 2001). ADM has been shown (Kubo *et al.*, 1998) to be overproduced by macrophages after inflammation and to modulate cytokine production (specifically TNF-*α*); several different independent studies support the fact that ADM is an anti-apoptotic peptide on different cell types (Bi *et al.*, 2007; Uzan *et al.*, 2006; Yin *et al.*, 2004). TNF-*α*IP3 is a cytoplasmic zinc finger protein that inhibits NF-*κβ* activity and TNF-mediated programmed cell death (Li *et al.*, 2006; Qin *et al.*, 2006). Downregulated genes included those encoding NLK, a stimulator of apoptosis (Yasuda *et al.*, 2003), HIF1-*α*, which has been suggested to favour apoptosis in the absence of oxygen (Bruick, 2000), and GRM5, known to protect neurons from apoptotic death (Maiese *et al.*, 2000). Taken together, these findings suggest that PRRSV actively induces an anti-apoptotic state in order to complete its virus replication cycle. This is discordant with previous results showing that PRRSV induces infected cells, as well as uninfected bystander cells, to undergo apoptosis (for examples, see Chang *et al.*, 2005; Sirinarumitr *et al.*, 1998), but it should be noted that those data were obtained with *in vitro* infection treatments much longer than 12 h. On the other hand, the absence of apoptotic induction by PRRSV has been observed in MARC-145 cells (Miller & Fox, 2004) and HeLa cells (Lee *et al.*, 2004). Interestingly, Kim *et al.* (2002) reported an atypical form of apoptosis that culminates in increased cell membrane permeability and late apoptosis after completion of virus replication.

The upregulation of *IL-10* gene expression (FC=1.9) indicates that the IL-10-mediated downregulation of the T-helper cell type 1 (Th1) response may be an important mechanism operated by PRRSV, as well as by other viruses (for reviews, see Fickenscher *et al.*, 2002; Redpath *et al.*, 2001). Upregulation of *IL-10* expression was found previously in PRRSV-infected porcine monocytes, macrophages and dendritic cells (Flores-Mendoza *et al.*, 2008; Suradhat *et al.*, 2003) and *in vivo* in PRRSV-infected pigs (Suradhat & Thanawongnuwech, 2003; Sutherland *et al.*, 2007; Thanawongnuwech & Thacker, 2003; Thanawongnuwech *et al.*, 2004). IL-10 in PRRSV-infected cells seems to be increased concurrent with the onset of viraemia and the development of clinical signs (Díaz *et al.*, 2005). Also, PIK3R1 (upregulated in this study: FC=1.6), is known to positively regulate the production of IL-10 (Saegusa *et al.*, 2007). These findings add to previous studies (Murtaugh *et al.*, 2002; Wang *et al.*, 2007), suggesting that PRRSV causes an imbalanced immune response characterized by an abundance of humoral immunity (Th2-mediated), which is less effective against viral pathogens.

The *TNF*-*α* gene was only slightly upregulated at 12 h p.i. (FC=1.5). The role of TNF-*α* in PRRSV infection is controversial: it has been reported that PRRSV is a potent inducer of TNF-*α* in PAMs at 18, 36, 54, 72, 90 and 108 h p.i. (Chang *et al.*, 2005) and at 6 and 15 h p.i. (Thanawongnuwech *et al.*, 2004). However, Charerntantanakul *et al.* (2006) showed that, in porcine monocytes infected by PRRSV, *IL-10* gene expression increased, and this response contributed to a reduction in TNF-*α* production. In fact, crucial anti-inflammatory activities of IL-10 may be due to its inhibitory effects on TNF-*α* production (Moore *et al.*, 2001). Overall, this suggests that IL-10 may also participate in fine-tuning the production and effects of TNF-*α*.

Other differentially expressed genes (see Table 1) confirmed that a complex pattern of TNF-*α* regulation takes place upon PRRSV infection. *TNF-αIP3*, known to be induced by TNF-*α* (Dixit *et al.*, 1990; Lee *et al.*, 2000) and suggested to protect against the inflammatory response to influenza virus infection (Onose *et al.*, 2006), was upregulated. *STAG2*, an enhancer of TNF production (Lara-Pezzi *et al.*, 2004), and the member of the MAPK pathway, *ATF2*, a transcription activator of both IFN-*β* and TNF-*α* in response to virus infection (Biron & Sen, 2001; Tsai *et al.*, 1996), were downregulated. The MAPK pathway was the most highly represented gene network identified in this study, with five differentially expressed genes at 12 h p.i. (*ATF2*, *TNF*-*α*, *MAP3K8*, *MKNK2* and *NLK*). The MAPK pathway is one of the most important pathways for immune response to infection (Bruder & Kovesdi, 1997; Yang *et al.*, 2007) and has been found to be modulated in PAMs after an antibody-mediated cross-linking treatment of sialoadhesin, the main PRRSV internalization receptor (Genini *et al.*, 2008), although in this case different genes of the pathway were involved.

In conclusion, this work has provided a genome-wide gene expression catalogue of PRRSV pathogenesis and has allowed us to picture how different genes and gene pathways are co-modulated in the physiological context of the PAM. As such, these results provide a large-scale and unbiased basis for further investigations on gene roles and functions. The sequencing of the pig genome currently in progress (www.piggenome.org), besides allowing a complete annotation of all transcripts, will soon give important clues for future genomics studies, for example for the interpretation of genome scan analyses aimed at identifying the genetic components involved in PRRSV resistance/susceptibility in swine populations (Ait-Ali *et al.*, 2007; Lewis *et al.*, 2007).

## Supplementary Material

[Supplementary Table]

## Figures and Tables

**Table 1. t1:** Transcripts differentially expressed in PAMs at 1, 3, 6, 9 and 12 h p.i. following PRRSV infection A total of 148 transcripts showed differential expression and are listed from the highest to the lowest fold change at the different time points p.i. The Affymetrix probe set IDs are reported with fold changes, gene symbols and gene description (Tsai *et al.*, 2006).

**Affymetrix probe set ID**	**Fold change**	**Gene symbol**	**Gene description**
**1 h p.i.**			
Ssc.10997.1.S1_at	1.699	*GRP58*	Protein disulfide-isomerase A3 precursor
Ssc.1313.1.A1_at	0.661	*NP_077001*	XTP3-transactivated protein A (*Homo sapiens*)
**3 h p.i.**			
Ssc.20199.2.S1_at	0.666	*HIVEP2*	Human immunodeficiency virus type I enhancer-binding protein 2
Ssc.30752.1.S1_at	0.659	*IFIT1*	IFN-induced protein with tetratricopeptide repeats 1
Ssc.23248.1.S1_at	0.653	*PTPRC*	Leukocyte common antigen precursor
AFFX-Ss_IRP_3_at	0.648	*IRG6*	*Sus scrofa* inflammatory response protein 6
Ssc.390.2.S1_at	0.550	*HIF1-α*	Hypoxia-inducible factor 1-*α*
**6 h p.i.**			
Ssc.12512.1.A1_at	1.544	*DDX17*	Probable RNA-dependent helicase p72 (DEAD-box protein 17)
Ssc.20344.1.S1_at	0.667	*WBP2*	WW domain-binding protein 2
Ssc.13400.2.S1_at	0.666	*C3orf4*	Protein C3orf4 (membrane protein GENX-3745) (HSPC174)
Ssc.8570.1.A1_at	0.665	*DLC1*	Rho-GTPase-activating protein 7
Ssc.13657.1.A1_at	0.663	*ATF2*	Cyclic-AMP-dependent transcription factor ATF-2
Ssc.16363.1.S1_at	0.662	*TMOD3*	Ubiquitous tropomodulin
Ssc.3420.1.S1_at	0.660	*C14orf111*	Protein C14orf111 (CGI-35)
Ssc.7164.1.A1_at	0.660	*NP_060530*	Mitochondrial isoleucine tRNA synthetase (*Homo sapiens*)
Ssc.22634.1.S1_at	0.654	*RB1CC1*	Rb1-inducible coiled coil protein 1 (*Homo sapiens*)
Ssc.1672.2.A1_at	0.652	*HNRPLL*	Heterogeneous nuclear ribonucleoprotein L-like
Ssc.16335.1.S2_at	0.651	*LPL*	Lipoprotein lipase precursor
Ssc.16422.2.A1_at	0.647	*PLAA*	Phospholipase A-2-activating protein
Ssc.30752.2.A1_at	0.645	*IFIT1*	IFN-induced protein with tetratricopeptide repeats 1
Ssc.2354.1.S1_at	0.637	*GPR160*	Probable G protein-coupled receptor 160
Ssc.17091.2.A1_s_at	0.637	*C3orf52*	TPA-induced transmembrane protein
Ssc.23248.1.S1_at	0.625	*PTPRC*	Leukocyte common antigen precursor
Ssc.1333.1.A1_at	0.611	*ZCCHC11*	Zinc finger, CCHC domain containing 11 isoform b
Ssc.26084.1.S1_at	0.607	*ATP2B1*	Plasma membrane calcium-transporting ATPase 1
Ssc.22221.2.S1_at	0.600	*GKP3*	Glycerol kinase, testis-specific 1
Ssc.13219.1.S1_at	0.599	*NP_689905*	Core 1 UDP-galactose : *N*-acetylgalactosamine-*α*-R *β*1,3-galactosyltransferase 2; core 1 *β*3-galactosyltransferase-specific molecular chaperone (*Homo sapiens*)
Ssc.19975.1.S1_at	0.592	*TEBP*	Telomerase-binding protein p23 (Hsp90 co-chaperone) (progesterone receptor complex p23) (*Homo sapiens*)
Ssc.4004.1.A1_at	0.577	*SYNE2*	Nesprin 2 (nuclear envelope spectrin repeat protein 2) (Syne-2) (synaptic nuclear envelope protein 2) (nucleus and actin connecting element protein) (NUANCE protein)
Ssc.10997.1.S1_at	0.535	*GRP58*	Protein disulfide-isomerase A3 precursor
Ssc.4472.1.A1_at	0.524	*NXT2*	NTF2-related export protein 2 (p15-2 protein) (DC9) (BM-025)
Ssc.390.2.S1_at	0.511	*HIF1-α*	Hypoxia-inducible factor 1-*α*
**9 h p.i.**			
Ssc.30752.2.A1_at	3.195	*IFIT1*	IFN-induced protein with tetratricopeptide repeats 1
Ssc.5085.1.A1_at	2.794	*TNF-αIP3*	Tumour necrosis factor, alpha-induced protein 3
Ssc.29006.1.S1_at	2.634	*IFN-β1*	IFN-*β* precursor
Ssc.30532.1.A1_at	2.546	*XRCC2*	DNA-repair protein XRCC2 (X-ray repair cross-complementing protein 2)
Ssc.286.1.S1_s_at	2.182	*cig5*	Viperin; similar to inflammatory response protein 6 (*Homo sapiens*)
AFFX-Ss_IRP_3_at	1.919	*IRG6*	*Sus scrofa* inflammatory response protein 6
Ssc.15761.1.A1_at	1.881	*TCRA*	T-cell receptor *α*-chain C region (*Homo sapiens*)
Ssc.314.1.S1_at	1.745	*ADM*	ADM precursor [contains adrenomedullin (AM)]
Ssc.29054.1.A1_at	1.639	*GBP1*	IFN-induced guanylate-binding protein 1
Ssc.14474.1.S1_at	1.538	*LOC396897*	*Sus scrofa* apomucin
Ssc.336.1.S1_at	1.521	*USP18*	Ubl carboxyl-terminal hydrolase 18
Ssc.11901.1.S1_at	0.652	*C10orf22*	Chromosome 10 open reading frame 22
Ssc.4462.1.S1_at	0.630	*SDHC*	Succinate dehydrogenase cytochrome b560 subunit, mitochondrial precursor (integral membrane protein CII-3)
Ssc.17091.2.A1_s_at	0.621	*C3orf52*	TPA-induced transmembrane protein
Ssc.5353.1.S1_at	0.620	*ZDHHC3*	Zinc finger DHHC domain containing protein 3 (zinc finger protein 373) (DHHC1 protein)
Ssc.15559.1.A1_s_at	0.552	*NP_060114*	DRE1 protein (*Homo sapiens*)
**12 h p.i.**			
Ssc.29006.1.S1_at	20.150	*IFN-β1*	IFN-*β* precursor
Ssc.30752.2.A1_at	4.917	*IFIT1*	IFN-induced protein with tetratricopeptide repeats 1
Ssc.30532.1.A1_at	4.079	*XRCC2*	DNA-repair protein XRCC2 (X-ray repair cross-complementing protein 2)
AFFX-Ss_IRP_3_at	3.827	*IRG6*	*Sus scrofa* inflammatory response protein 6
Ssc.286.1.S1_s_at	3.778	*cig5*	Viperin; similar to inflammatory response protein 6 (*Homo sapiens*)
Ssc.5085.1.A1_at	2.912	*TNF-αIP3*	Tumour necrosis factor-*α*-induced protein 3
Ssc.15761.1.A1_at	2.689	*TCR-α*	T-cell receptor *α*-chain C region (*Homo sapiens*)
Ssc.336.1.S1_at	2.034	*USP18*	Ubl carboxyl-terminal hydrolase 18
Ssc.148.1.S1_at	1.934	*IL-10*	Interleukin-10 precursor
Ssc.12284.1.A1_at	1.923	*SGK*	Serine/threonine-protein kinase Sgk1
Ssc.11048.1.S1_at	1.918	*PLAC8*	Placenta-specific gene 8 protein
Ssc.16288.1.S1_at	1.859	*IGHM*	Ig *α*-1 chain C region
Ssc.29054.3.S1_at	1.738	*GBP1*	IFN-induced guanylate-binding protein 1
Ssc.314.1.S1_at	1.718	*ADM*	ADM precursor [contains adrenomedullin (AM)]
Ssc.18038.1.A1_at	1.642	*MAP3K8*	Mitogen-activated protein kinase kinase kinase 8
Ssc.10754.1.A1_at	1.621	*PIK3R1*	Phosphatidylinositol 3-kinase regulatory *α* subunit (PI3-kinase p85-alpha subunit)
Ssc.26507.2.S1_at	1.585	*NP_073596*	Endo-*β*-*N*-acetylglucosaminidase (*Homo sapiens*)
Ssc.25855.1.S1_at	1.531	*XP_846553*	PREDICTED: hypothetical protein
Ssc.1701.2.S1_at	1.529	*Q6PK96*	Cytochrome *b*, ascorbate-dependent 3
Ssc.100.1.S1_at	1.513	*TNF-α*	Tumour necrosis factor precursor (TNF-*α*)
Ssc.13657.1.A1_at	0.665	*ATF2*	Cyclic-AMP-dependent transcription factor ATF-2 (activating transcription factor 2)
Ssc.4498.1.S1_at	0.663	*IXL*	Intersex-like
Ssc.3420.1.S1_at	0.661	*C14orf111*	Protein C14orf111 (CGI-35)
Ssc.8311.1.A1_at	0.660	*MPI*	Mannose-6-phosphate isomerase
Ssc.8541.1.A1_at	0.659	*STAG2*	Cohesin subunit SA-2 (Stromal antigen 2)
Ssc.16422.2.A1_at	0.658	*PLAA*	Phospholipase A-2-activating protein
Ssc.21796.1.S1_at	0.657	*SORL1*	Sortilin-related receptor precursor (sorting protein-related receptor containing LDLR class A repeats)
Ssc.5404.1.S1_at	0.657	*MOSPD1*	Motile sperm domain-containing 1 (*Homo sapiens*)
Ssc.27060.1.A1_at	0.656	*SSSCA1*	Sjogren's syndrome/scleroderma autoantigen 1 (autoantigen p27).
Ssc.26553.1.S1_at	0.655	*AP1-γ1*	Adaptor-related protein complex 1 *γ*1 subunit (*γ*-adaptin) (adaptor protein complex AP-1 *γ*1 subunit)
Ssc.3656.1.S1_at	0.654	*KHLX*	Kelch-like protein X (*Homo sapiens*)
Ssc.24239.1.S1_at	0.653	*C10orf45*	PREDICTED: *Pan troglodytes* hypothetical protein LOC737061
Ssc.9314.2.S1_at	0.653	*TP53RK*	TP53 regulating kinase
Ssc.17314.1.S1_at	0.653	*C3orf10*	Probable protein BRICK1
Ssc.16057.2.S1_a_at	0.650	*GANC*	Calpain 3 (EC 3.4.22.-) (Calpain L3)
Ssc.2756.1.A1_at	0.650	*MRPL22*	Mitochondrial ribosomal protein L22 (*Homo sapiens*)
Ssc.26084.1.S1_at	0.649	*ATP2B1*	Plasma membrane calcium-transporting ATPase 1
Ssc.8283.1.A1_at	0.649	*PTPN11*	Protein-tyrosine phosphatase, non-receptor type 11
Ssc.26735.1.A1_at	0.649	*Q96BP3*	Peptidylprolyl isomerase domain and WD repeat containing 1 (*Bos taurus*)
Ssc.8430.1.A1_at	0.649	*Q86W74*	Ankyrin repeat domain 46 (ANKRD46) (*Bos taurus*)
Ssc.1441.1.S1_at	0.649	*DCTN3*	Dynactin 3 isoform 1; dynactin light chain (*Homo sapiens*)
Ssc.10542.1.S1_at	0.647	*EXOSC1*	3′→5′ ExoRNase CSL4 homologue
Ssc.772.1.S1_at	0.643	*CARHSP1*	Calcium-regulated heat-stable protein 1
Ssc.3281.1.S1_at	0.643	*C11orf10*	UPF0197 protein C11orf10 (HSPC005)
Ssc.16495.1.A1_at	0.643	*DF5L*	Gasdermin domain containing protein 1 (*Homo sapiens*)
Ssc.4306.1.A1_at	0.642	*MESDC1*	Mesoderm development candidate 1
Ssc.26318.1.S1_at	0.638	*DNCL1*	Dynein light chain 1, cytoplasmic
Ssc.24811.1.A1_at	0.638	*Q6NSI4*	Nuclear receptor-binding protein 2 (NRBP2) (*Bos taurus*)
Ssc.1153.1.A1_at	0.638	*C9orf28*	C9orf28 protein
Ssc.22634.1.S1_at	0.637	*RB1CC1*	Rb1-inducible coiled-coil protein 1 (*Homo sapiens*)
Ssc.19778.1.S1_at	0.633	*TNF-αIP8L2*	Tumour necrosis factor-*α*-induced protein 8-like 2 (*Bos taurus*)
Ssc.8604.1.A1_at	0.631	*SNX24*	Sorting nexin 24 (SBBI31)
Ssc.5353.1.S1_at	0.627	*ZDHHC3*	Zinc finger DHHC domain-containing protein 3
Ssc.3154.1.S1_at	0.627	*GRM5*	Metabotropic glutamate receptor 5 precursor (mGluR5)
Ssc.6833.1.S1_at	0.625	*BTG1*	B-cell translocation protein 1 (*Homo sapiens*)
Ssc.1160.1.S1_at	0.624	*PSMC3*	26S protease regulatory subunit 6A (TAT-binding protein 1) (TBP-1) (proteasome subunit P50)
Ssc.12944.1.A1_at	0.623	*RPA3*	Replication protein A 14 kDa subunit
Ssc.10037.1.A1_at	0.622	*NLK*	Serine/threonine kinase NLK
Ssc.22120.1.S1_a_at	0.617	*RYR2*	PREDICTED: similar to RIKEN cDNA 3110009E18 (*Homo sapiens*)
Ssc.18253.1.S1_at	0.616	*F8*	Coagulation factor VIII precursor
Ssc.24739.1.A1_at	0.616	*SLC16A7*	Monocarboxylate transporter 2 (MCT 2)
Ssc.16936.2.S1_a_at	0.615	*Q9P0T8*	Similar to hypothetical protein HSPC111 (*Bos taurus*)
Ssc.16691.1.S1_at	0.609	*H2AF-J*	H2A histone family, member J isoform 1 (*Homo sapiens*)
Ssc.30182.1.A1_at	0.607	*RER1*	RER1 protein (*Homo sapiens*)
Ssc.21559.1.S1_at	0.606	*ANKRD10*	Ankyrin repeat domain protein 10
Ssc.11369.1.S1_at	0.605	*NP_077271*	Derlin-1 (Der1-like protein 1)
Ssc.1029.1.S1_at	0.603	*PHF6*	PHD finger protein 6 (PHD-like zinc finger protein)
Ssc.13954.1.A1_at	0.602	*Q5VV17*	PREDICTED: similar to hypothetical protein DKFZp761A052
Ssc.11878.1.S1_at	0.601	*HMBS*	Porphobilinogen deaminase
Ssc.16677.1.S1_a_at	0.599	*C17orf37*	Uncharacterized protein C17orf37 (protein C35) (HBV X-transactivated gene 4 protein)
Ssc.24943.1.S1_at	0.597	*NDUFA11*	NADH-ubiquinone oxidoreductase subunit B14.7
Ssc.3426.1.A1_at	0.595	*MAPK6*	Mitogen-activated protein kinase 6
Ssc.21783.1.S1_at	0.595	*MRPL2*	Mitochondrial ribosomal protein L2 (*Homo sapiens*)
Ssc.13370.1.A1_at	0.595	*Q8NA66*	RIKEN cDNA 1810054D07 gene (1810054D07Rik) (*Mus musculus*)
Ssc.1206.1.A1_at	0.595	*ADAMTS19*	ADAMTS-19 precursor
Ssc.6979.1.A1_at	0.587	*TPP2*	Tripeptidyl-peptidase II
Ssc.13218.1.A1_at	0.587	*NP_660155*	Testis development protein NYD-SP29 (*Homo sapiens*)
Ssc.19975.1.S1_at	0.582	*TEBP*	Telomerase-binding protein p23 (Hsp90 co-chaperone) (*Homo sapiens*)
Ssc.16392.2.A1_a_at	0.574	*MKNK2*	MAP kinase-interacting serine/threonine kinase 2
Ssc.6230.2.A1_at	0.573	*SDCCAG3*	Serologically defined colon cancer antigen 3 (*Homo sapiens*)
Ssc.21987.1.A1_at	0.57	*IFRD1*	IFN-related developmental regulator 1
Ssc.5163.1.S1_at	0.569	*GCNT2*	*N*-Acetyllactosaminide *β*-1,6-*N*-acetylglucosaminyl-transferase
Ssc.6189.1.A1_at	0.565	*SLC7A11*	Cystine/glutamate transporter (amino acid transport system x_c_^−^)
Ssc.1333.1.A1_at	0.565	*ZCCHC11*	Zinc finger, CCHC domain containing 11 isoform b
Ssc.29047.1.S1_at	0.561	*HIG2*	Hypoxia-inducible protein 2 (*Homo sapiens*)
Ssc.22287.1.S1_at	0.561	*GABR-α3*	*γ*-Aminobutyric-acid receptor *α*-3 subunit precursor [GABA(A) receptor]
Ssc.2354.1.S1_at	0.554	*GPR160*	Probable G protein-coupled receptor 160
Ssc.4004.1.A1_at	0.552	*SYNE2*	Nesprin 2 (nuclear envelope spectrin repeat protein 2)
Ssc.13400.2.S1_at	0.550	*C3orf4*	Protein C3orf4 (membrane protein GENX-3745)
Ssc.26309.1.A1_at	0.548	*CHES1*	Checkpoint suppressor 1 (Forkhead box protein N3)
Ssc.6513.1.S1_at	0.542	*LRRC28*	Leucine-rich repeat-containing 28 (*Homo sapiens*)
Ssc.3451.1.S1_at	0.540	*SLC11A2*	Natural resistance-associated macrophage protein 2 (NRAMP2)
Ssc.4462.1.S1_at	0.536	*SDHC*	Succinate dehydrogenase cytochrome b560 subunit, mitochondrial precursor (integral membrane protein CII-3)
Ssc.14114.1.A1_at	0.525	*ABCD3*	ATP-binding cassette, subfamily D, member 3 (70 kDa peroxisomal membrane protein) (PMP70)
Ssc.16563.1.S1_at	0.513	*NP_067050*	DC2 protein (*Homo sapiens*)
Ssc.1527.1.A1_at	0.503	*SLC20A1*	Solute carrier family 20 (phosphate transporter), member 1; Glvr-1; PiT-1; gibbon ape leukemia virus receptor 1 (*Homo sapiens*)
Ssc.16475.1.S1_at	0.475	*COL4-α3*	Collagen *α*3(IV) chain precursor (Goodpasture antigen)
Ssc.17091.2.A1_s_at	0.463	*C3orf52*	TPA-induced transmembrane protein
Ssc.4472.1.A1_at	0.459	*NXT2*	NTF2-related export protein 2 (p15-2 protein) (DC9) (BM-025)
Ssc.15559.1.A1_s_at	0.306	*NP_060114*	DRE1 protein (*Homo sapiens*)

**Table 2. t2:** GO analysis and ranking of the 100 transcripts differentially expressed at 12 h p.i Ranking and assignment is given for the differentially expressed transcripts at 12 h p.i. to the three GO categories: biological process, molecular function and cellular component. The number of transcripts for each process is shown, with the corresponding *e*-values.

**GO category**	**Ranking**	**Number of transcripts**	***e*-value**
**Biological process**			
Response to stimulus	1	22	6.50×10^−4^
Response to stress	2	14	6.10×10^−3^
Immune response	3	11	6.60×10^−3^
Physiological process	4	68	8.30×10^−3^
Defence response	5	11	1.20×10^−2^
Response to biotic stimulus	6	11	1.60×10^−2^
Anion transport	7	5	2.00×10^−2^
Regulation of apoptosis	8	7	2.30×10^−2^
Regulation of programmed cell death	9	7	2.40×10^−2^
Meiosis	10	3	3.00×10^−2^
M phase of meiotic cell cycle	11	3	3.00×10^−2^
Macromolecule metabolism	12	34	3.10×10^−2^
Meiotic cell cycle	13	3	3.10×10^−2^
Organismal physiological process	14	16	3.60×10^−2^
Anti-apoptosis	15	4	4.30×10^−2^
M phase	16	5	4.40×10^−2^
Inorganic anion transport	17	4	5.30×10^−2^
Response to virus	18	3	5.40×10^−2^
Negative regulation of apoptosis	19	4	6.00×10^−2^
Negative regulation of programmed cell death	20	4	6.10×10^−2^
Apoptosis	21	8	6.20×10^−2^
Programmed cell death	22	8	6.30×10^−2^
Activation of NF-*κβ* transcription factor	23	2	6.40×10^−2^
Response to wounding	24	6	7.00×10^−2^
Cell death	25	8	7.30×10^−2^
Death	26	8	7.50×10^−2^
Protein metabolism	27	25	7.60×10^−2^
Negative regulation of cell proliferation	28	4	7.80×10^−2^
Positive regulation of transcription factor activity	29	2	7.90×10^−2^
Leukocyte adhesion	30	2	7.90×10^−2^
B-cell proliferation	31	2	7.90×10^−2^
Negative regulation of cellular process	32	9	8.30×10^−2^
Interaction between organisms	33	3	8.60×10^−2^
Ion transport	34	8	9.20×10^−2^
**Molecular function**			
Antigen binding	1	3	1.50×10^−2^
Receptor binding	2	8	6.00×10^−2^
Phosphatase binding	3	2	8.70×10^−2^
Tumour necrosis factor receptor binding	4	2	8.70×10^−2^
Cytokine activity	5	4	8.80×10^−2^
**Cellular component**			
Integral to membrane	1	26	6.90×10^−2^
Intrinsic to membrane	2	26	7.10×10^−2^
Extracellular space	3	6	8.80×10^−2^

**Table 3. t3:** Real-time PCR results of genes differentially expressed following PRRSV infection of PAMs Real-time PCR results of ten selected genes in two pools of PAMs infected with PRRSV (I) compared with control PAMs (C). The reported values are the means±sd of technical triplicates and were calculated as described in Methods; values that significantly differ between infected and control PAMs are indicated in bold (*, *P*<0.10; **, *P*<0.05). The last column gives the Pearson's correlation coefficient (*r*) between real-time and microarray data.

**Gene symbol**	**Status**	**Pool I**					**Pool II**					***r***
		**1 h**	**3 h**	**6 h**	**9 h**	**12 h**	**1 h**	**3 h**	**6 h**	**9 h**	**12 h**	
*IFN-β*	I	0.01±0.005	0.04±0.016*	0.01±0.006	0.13±0.032**	2.03±0.402**	0.01±0.003	0.05±0.011*	0.03±0.005	0.17±0.032**	2.76±0.556**	0.95
	C	0.02±0.008	0.01±0.005*	0.004±0.002	0.01±0.002**	0.01±0.006**	0.01±0.005	0.01±0.007*	0.03±0.013	0.02±0.006**	0.03±0.01**	
*TNF-α*	I	4.02±1.614	1.37±0.467	0.3±0.106	0.25±0.072	1.16±0.425	5.95±2.325	1.76±0.679	0.59±0.102	0.89±0.236	2.55±0.728**	0.94
	C	7.52±2.786	0.99±0.28	0.32±0.088	0.16±0.05	2.09±0.598	5.89±2.119	1.45±0.462	0.62±0.221	0.41±0.139	0.36±0.138**	
*TNF-αIP3*	I	2.1±0.854	0.59±0.2	0.32±0.039	0.75±0.203**	1.31±0.4*	4.01±2.172	1.19±0.461	0.44±0.09	0.81±0.16**	3.17±1.232*	0.94
	C	1.76±0.484	0.33±0.061	0.22±0.035	0.2±0.026**	0.42±0.12*	3.59±1.403	0.81±0.255	0.47±0.137	0.33±0.078**	0.24±0.071*	
*USP18*	I	0.25±0.085	0.41±0.125	0.78±0.12	0.92±0.166*	1.61±0.393**	0.34±0.12	0.69±0.227	1.06±0.221	1.25±0.205*	2.37±0.868**	0.88
	C	0.27±0.079	0.3±0.036	0.74±0.121	0.54±0.038*	0.53±0.106**	0.29±0.069	0.45±0.132	0.93±0.195	0.52±0.14*	0.44±0.123**	
*cig5*	I	0.13±0.019	0.48±0.065	0.97±0.183	0.91±0.083*	1.72±0.102**	0.15±0.021	1.16±0.290	1.15±0.192	1.33±0.274**	2.37±0.137**	0.89
	C	0.14±0.019	0.50±0.102	0.79±0.151	0.58±0.123*	0.86±0.258**	0.11±0.031	0.88±0.116	0.90±0.180	0.40±0.054**	0.45±0.164**	
*IL-10*	I	0.62±0.228	0.52±0.199	0.19±0.094	0.49±0.151	1.6±0.415*	1.08±0.246	0.68±0.14	0.38±0.074	0.67±0.166	2.05±0.567**	0.88
	C	0.57±0.107	0.18±0.087	0.18±0.047	0.23±0.04	0.47±0.06*	1.17±0.294	0.43±0.119	0.45±0.196	0.44±0.091	0.7±0.177**	
*GRP58*	I	1.92±0.103**	0.99±0.175*	0.66±0.144**	1.82±0.248	2.22±0.307**	1.22±0.187	1.55±0.195*	1.28±0.088	0.66±0.042**	1.13±0.147	0.09
	C	1.00±0.046**	1.61±0.179*	1.49±0.268**	1.19±0.161	0.66±0.043**	1.31±0.166	0.92±0.123*	1.40±0.183	1.29±0.154**	1.16±0.126	
*IFN-α*	I	0.07±0.013	0.18±0.028**	0.03±0.002	0.09±0.012	0.13±0.020	0.05±0.002	0.16±0.023**	0.14±0.018	0.09±0.005**	0.14±0.022	–
	C	0.08±0.015	0.03±0.002**	0.03±0.002	0.06±0.013	0.08±0.013	0.05±0.004	0.04±0.004**	0.11±0.007	0.04±0.005**	0.11±0.004	
*IFN-αR1*	I	0.93±0.274	0.96±0.239	0.91±0.165	0.85±0.187	0.87±0.25	1.01±0.139	1.06±0.115	1.10±0.035	1.19±0.10	0.95±0.151	–
	C	1.11±0.450	1.096±0.265	1.12±0.220	0.84±0.202	1.02±0.192	1.06±0.129	1.06±0.114	0.96±0.133	1.20±0,128	1.06±0.058	
*Sialoadhesin*	I	0.25±0.016**	0.17±0.019	0.14±0.009	0.19±0.026	0.22±0.034	0.19±0.017	0.19±0.016	0.21±0.010	0.27±0.048*	0.46±0.071*	–
	C	0.17±0.033**	0.16±0.018	0.15±0.005	0.23±0.051	0.15±0.024	0.18±0.008	0.21±0.015	0.22±0.020	0.18±0.025*	0.28±0.010*	
