# Supplementary material for: Genome-wide transcriptional response of primary alveolar macrophages following infection with porcine reproductive and respiratory syndrome virus
Source: J Gen Virol. 2008 Oct;89(Pt 10):2550–64. doi: 10.1099/vir.0.2008/003244-0 (PMC2885007; doi:10.1099/vir.0.2008/003244-0)
Supplement: [Supplementary Table] [file supp_89_10_2550__index.html]

 Genome-wide transcriptional response of primary alveolar macrophages following infection with porcine reproductive and respiratory syndrome virus -- Genini et al. 89 (10): 2550 Data Supplement - Supplementary Table -- Journal of General Virology

## Supplementary Data

### Genome-wide transcriptional response of primary alveolar macrophages following infection with porcine reproductive and respiratory syndrome virus (PRRSV), by Sem Genini, Peter L. Delputte, Roberto Malinverni, Maria Cecere, Alessandra Stella, Hans J. Nauwynck and Elisabetta Giuffra

*Journal of General Virology* vol. **89**, part 10, pp. 2550–2564

**Supplementary Table S1.**  List of genes and primers analysed by real-time PCR [PDF] (56 KB)

  
  
