# Supplementary material for: Genome-wide transcriptional response of primary alveolar macrophages following infection with porcine reproductive and respiratory syndrome virus
Source: J Gen Virol. 2008 Oct;89(Pt 10):2550–64. doi: 10.1099/vir.0.2008/003244-0 (PMC2885007; doi:10.1099/vir.0.2008/003244-0)
Supplement: [Supplementary Table] [file supp_89_10_2550__1.pdf]

Supplementary Table S1. List of genes and primers analysed by real-time PCR

Gene symbols are reported together with the Affymetrix ID, the category number of the Roche Probe library and the primer sequences.

**Gene symbol**	**Affymetrix ID**	**Roche Universal Probe Library (probe no.)**	**Forward primer (5′→3′)**	**Reverse primer (5′→3′)**
IFN-β	Ssc.29006.1.S_at	25	GGAGACAATCCTGGAGGAAAT	TTCAGGTGAAGAATGGTCATGT
cig5	Ssc.286.1.S1_s_at	14	TCAAAAGTTTGGTTTGTGTGATTT	TGCCTGACAATGATTCTGTTG
TNF-α	Ssc.100.1.S1_at	5	GGATGCAACCTGGGACAT	ATTCCGATTGGAACCCAAG
TNF-α*IP3*	Ssc.5085.1.A1_at	21	CGCTGCTGGAGAACAGTTT	AGAATCTCACGCTTCTTCGTG
IL-10	Ssc.148.1.S1_at	16	ACCCGGGAAAGCTAGTGG	GCCCAGGGACAAGAGAAAG
USP18	Ssc.336.1.S1_at	71	GACCTTGTGCAGGTCTGGAT	GGCTCAGGACCTGCTAACC
GRP58	Ssc.10997.1.S1_at	83	CCCTGAAGATATTTAGAGATGGTGA	TGGACCAGCCTGCTTCTT
IFN-α	Ssc.19264.1.S1_at	66	TCCAGCTCTTCAGCACAGAG	AGCTGCTGATCCAGTCCAGT
IFN-α*R1*	Ssc.11381.1.S1_at	27	TGGAAGCAAAATAGGAGAAGAAA	CCCAAGATTTTAAAGCGTTCTG
Sialoadhesin	–	66	ACCAGCCACATGGAGACC	GGTGAATCTCCTTCTGCATCC
ORF7 (PRRSV)	–	42	CAATCAAGGCGCAGGAAC	CGGCAGCATAAACTCAACCT
HPRT1	–	66	TGAGAAGCCGCATTTTCC	TCGGTCTGAGTGAGGTGATG
